# Board interlocks and Corporate Social Responsibility data in the Mexican Stock Exchange

**DOI:** 10.1016/j.dib.2022.108231

**Published:** 2022-05-04

**Authors:** Arturo Briseño-García

**Affiliations:** Universidad Autónoma de Tamaulipas, Facultad de Comercio y Administración Victoria, Matamoros S/N Zona Centro, Cd. Victoria, Tamaulipas, México

**Keywords:** Homophily, Practice Adoption, Mimetic, Empresa Socialmente Responsable, Markov Clusters, CEO Duality, CEO Power

## Abstract

Board interlocks (i.e. individuals that participate in two or more board positions) are a common mechanism to understand the network configuration of firms [2]. This paper presents and describes 2011 network and Corporate Social Responsibility (CSR) data using content analysis from annual reports of 137 listed firms in the Mexican Stock Exchange. [1]. Board interlocks were identified using board member names from each firm, creating a board member's list and matching them with the corresponding firm, resulting in a *nxn* matrix with 518 board interlocks. *Empresa Socialmente Responsable* variable was obtained from the 2012 list published every year by *Centro Mexicano para la Filantropía,* the non-for-profit organization in Mexico evaluating self-reporting CSR practices. Other variables were collected from firm's annual reports and Bloomberg's financial database. Finally, network characteristics such as the firm's centrality were calculated using UCINET 6.

## Specifications Table

**Table:** 

Subject	Business Management and Decision Sciences
Specific subject area	Strategy and Management
Type of data	Network matrices
How the data were acquired	The data was acquired using three different sources. First, the Centro Mexicano para la Filantropía website where publish a list of firms awarded with the Empresa Socialmente Responsible distinction every year. The Second Source is the Mexican Stock Exchange where the data was acquired via content analysis from the firm's annual reports. Finally, financial data was acquired by accessing the Bloomberg database.
Data format	Raw
Description of data collection	Listed firms in Mexico are generally large firms from diverse industries representing a considerable geographic dispersion on the Mexican territory. For this data collection, the sample of listed firms consists of those participating exclusively in the stock market and only for those participating in 2011. As a result, this data set represents not a sample of listed firms but all listed firms from that year. However, some firms were discarded from this data, especially those which are government-related.
Data source location	• Institution*: Centro Mexicano para la Filantropía* (Cemefi) website (https://www.cemefi.org/) • Country: México • Institution*:* Mexican Stock Exchange website (https://www.bmv.com.mx/) • Country: México • Institution*:* Bloomberg • Country: México
Data accessibility	Repository name: Mendeley data Data identification number: Version 1 Direct URL to data: https://data.mendeley.com/datasets/d8s2jg5n6t/1
Related research article	A. Briseño-García, B. W. Husted, & E. Arango-Herera, Do birds of a feather certify together? The impact of board interlocks on CSR certification homophily (2022). *J Bus Res*, 144, 336-344. https://doi.org/10.1016/j.jbusres.2022.01.080

## Value of the Data

• Data on board interlocks contains information about social ties formed by board members that participate in two or more firms [3]. While databases on board interlocks are more commonly available for developed regions, it is not the case for emerging ones. In this context, interlocking directorates data is scarce, dispersed, or even non-existent [4]. This data set on the Mexican context can provide the opportunity to increase Latin American/emerging economies studies on board interlocks and network characteristics to test theory traditionally documented in developed countries.
• The two-mode network provided in this dataset increases the possibilities to further analyze board interlocks. A two-mode network, i.e. a matrix arrangement of individuals (rows) and firms (columns) allows identifying board members individually and quantifying variables such as the number of board members for each firm.
• Management scholars frequently seek into other sciences and disciplines to explain organizational phenomena. This is the case for social networks theory, a set of concepts and methods to understand social phenomena commonly used in different areas such as sociology, anthropology, or health sciences. Accordingly, management researchers can use network data based on board interlocks to expand explanations on organizational topics such as practice adoption, environmental performance, decision-making process, leadership, among others.
• This data set is a cross-sectional representation of the network formed by interlocking directors in Mexico. Further studies can add data to test hypotheses using a longitudinal network to strengthen the conclusions drawn from data around organizational topics. Also, further insights can compare across countries. Board interlocks can vary their influence according to their context representing an opportunity for comparative studies.

## 1 Data Description

The dataset that appears in this article includes network matrices that represent a firm's similarity around characteristics such as industry, CEO duality, or profitability as well as the board interlocks’ ties (i.e. two firms sharing one or more board members). This data was used in Briseño et al.,(2022) to provide evidence on how social networks influence adoption of practices. In particular, the paper aims to identify among mimetic normative and coercive institucional mechanisms influencing the adioption of Corporate Social Responsibility practices. The study finds that firms are influenced by mimetism via corporate connections from those firms that shared board members (board interlocks), rather than normative (industry) or coercive (business groups) influences.

The present article provides further insights on the network influences including details on board across listed firms in Mexico. Aditionally, the dataset in this paper provides the opportunity for future research to calculate and include additional network variables not considered in the original article such as betweenes centrality or structural equivalence.

**Fig. 1:**
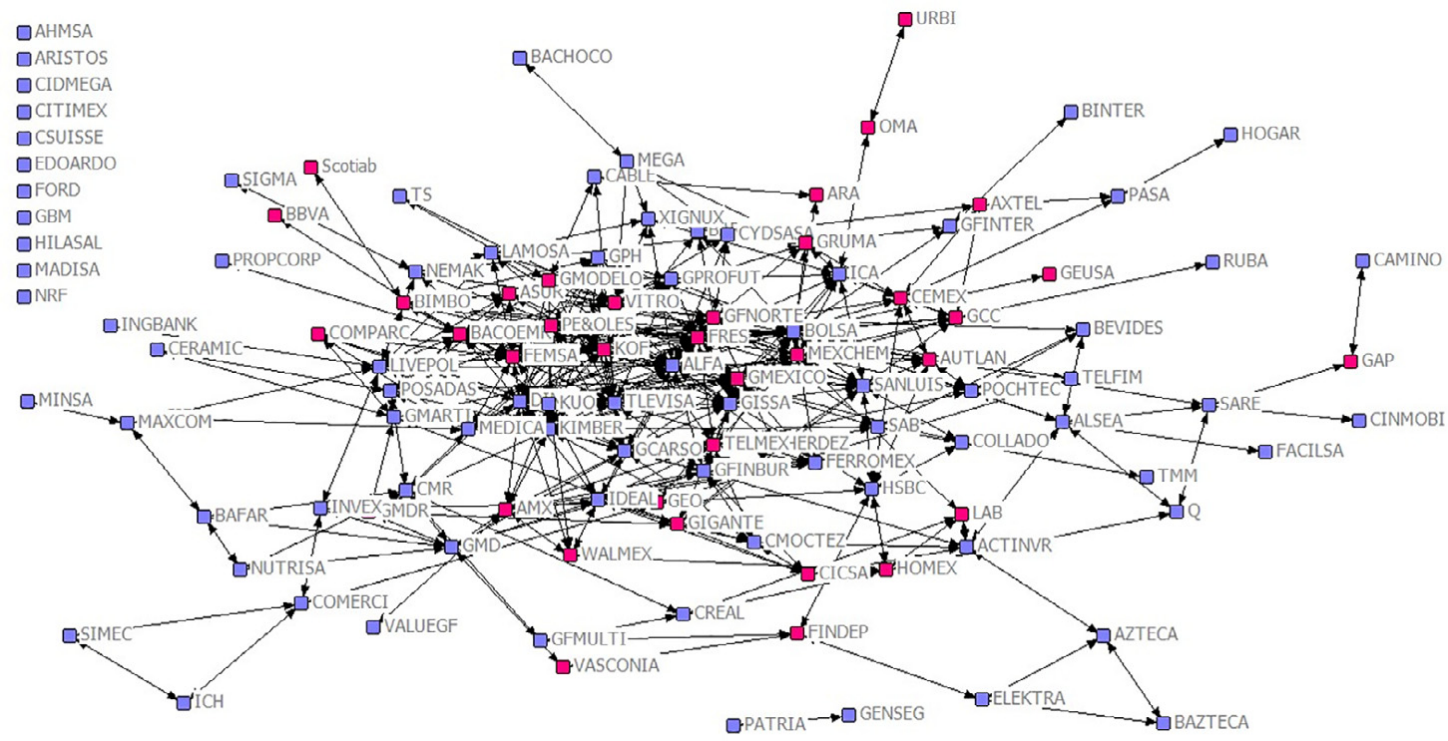
Board interlock network of firms listed in the Mexican Stock Exchange in 2011.

Concordantly, the data in this paper consists of ten matrices from 137 listed firms in 2011 (except for the ESR certification which refers to 2012). The interlock matrix can be visualized in Fig. 1.

Also, Fig. 1 shows in red color those listed firms that are included in the annual list created by *Centro Mexicano para la Filantropia (CEMEFI)* in 2012. This network is illustrative of the relationships created by board interlocks and the distribution in the network of firms engaging in social and environmental activities regarding the ESR certification by CEMEFI.

Table 1 presents additional information on descriptive statistics for this data set. For example, the maximum number of board interlocks for a listed firm in this study is 26 while there are firms that have no interlocks at all and are isolated in the network. Other interesting information is that 31% of listed firms appear in the ESR certification list while 38% of listed firms have the CEO also like the board president.

**Table 1:** Descriptive statistics of variables in this dataset

	ESR_2012	Board interlocks	Business group	Assets	ROA	Reach Centrality	CEO Duality
Mean	0.31	5.81	0.24	69290.46	3.44	0.25	0.38
Median	0	4	0	21703	1.965	0.286	0
STD	0.46	6.11	0.43	149802.28	43.98	0.14	0.49
Min	0	0	0	580	-415.388	0.007	0
Max	1	26	1	950000	228.211	0.448	1

Table 2 further describes the data set containing frequencies and intervals of the main variables. For example, the distribution of listed firms in terms of Industry, Business Groups, or Markov Clusters, provide an idea on how this group of listed firms are represented.

**Table 2:** Descriptive information on variables.

ESR_2012	N° of Firms	Industry	N° of Firms	Business group	N° of Firms	Assets	N° of Firms
0	95	Energy	0	0	104	No data	42
1	42	Construction materials	20	1	33	< 100k	80
		Industrial	30			100k < X < 200k	8
		Service and non-basic goods	15			200k < X < 300k	2
		Frequently consumed products	19			300k < X < 400k	2
		Health	5			400k < X < 500k	0
		Financial services	39			500k < X < 600k	1
		Information Technologies	0			600k < X < 700k	0
		Telecommunication services	9			700k < X < 800k	0
		Public Services	0			800k < X < 900k	1
						900k < X < 1000k	1
Total	137		137		137		137

ROA	N° of Firms	Clusters	N° of Firms	Reach Centrality	N° of Firms	CEO Duality	N° of Firms

X < 0	16	1	30	X < 0.10	29	No data	1
X = 0	26	2	27	0.10 < X < 0.20	4	0	84
0 < X < 10	77	3	7	0.20 < X < 0.30	42	1	52
10 < X < 20	14	4	2	0.30 < X < 0.40	51		
20 < X < 30	1	5	47	0.40 < X < 0.50	11		
30 < X < 40	1	6	4				
X > 50	2	7	7				
		8	4				
		9	3				
		10	2				
		11	2				
		12	2				
	137		137		137		137

## 2 Experimental Design, Materials and Methods

The dataset in this paper consists of a collection of variables intended to measure the relational aspects of listed firms in Mexico. In the next paragraphs, I explain how each variable was obtained and transformed.

The ESR2012 matrix consists of certified listed firms in 2012. The information was retrieved from the Centro Mexicano para la Filantropía website where they publish a list with firms awarded with the Empresa Socialmente Responsible distinction every year. This list was then transformed into an nxn matrix using UCINET 6. The method followed was the “attribute to matrix” conversion and the “product” option.

The TwomodeInterlocks2011 matrix consists of firms that share at least one board member in 2011. The data were acquired by content analysis of the annual reports that the Mexican Stock Exchange website provides in the section “digital information”. I retrieved the names of the board members for each firm producing a two-mode network, i.e. a matrix arrangement where rows are board members and columns are listed firms. The Interlocks2011 matrix was generated with the TwomodeInterlocks2011. This two-mode matrix is converted into a one-mode using UCINET 6 and the Affiliation 2-mode to 1-mode option. This command generates an nxn matrix with firms in both rows and columns. Then, the matrix consists of each combination of two pairs of firms where 1 represents the presence of an interlock and 0 the absence of this relationship.

The industry matrix is generated using the Mexican Stock Exchange classification available on its website. The resulting list of firms, with its corresponding industry, was transformed into an nxn matrix using UCINET 6. The method followed was the “attribute to matrix” conversion and the “exact match” option. Business Group variable was generated by content analysis of annual reports. Firms that belong to the same business group are classified. This attribute list was transformed into an nxn matrix using UCINET 6. The method followed was the “attribute to matrix” conversion and the “exact match” option.

Assets matrix was created by using the value of the firm's assets in 2011. The monetary value was retrieved from Bloomberg for each listed firm in that year. The nxn matrix was created using UCINET 6. The method followed was the “attribute to matrix” conversion and the “identity coefficient” option. ROA matrix was created by using the Return on Assets ratio of the firm in 2011. The score was retrieved from Bloomberg for each listed firm in that year. The nxn matrix was created using UCINET 6. The method followed was the “attribute to matrix” conversion and the “identity coefficient” option.

Clusters variable represents the region in the network in which each firm is positioned. This variable is created using UCINET 6 and the subgroup category. With the Interlocks2011 matrix as the network, the Clusters nxn matrix is created using “Markov Clustering” algorithm.

Another network variable in this dataset is reach centrality, which represents the number of “steps” that a firm must take to reach another firm in the network. This variable is created using UCINET 6 and the subgroup category. With the Interlocks2011 matrix as the network, the Reach nxn matrix is created using “Reach Centrality” option.

CEO duality variable represents the nxn matrix of two firms sharing a similar characteristic, i.e. the CEO and the Board President are the same person, this characteristic is known as CEO duality in the business literature. This variable is created using UCINET 6. With the Interlocks2011 matrix as the network, the Reach nxn matrix is created using “Reach Centrality” option. Finally, the CEO power variable represents the nxn matrix of two firms sharing similar levels of CEO power. The power of the CEO is estimated using those CEO that is the president of the board and participates in a firm that is central in the network. This combination reflects how influential the CEO might be both internally to the firm and externally in the network.

The nxn CEO power matrix is then, the product of two matrices, i.e. CEO duality and Freeman´s closeness centrality calculated using UCINET 6.

## CRediT Author Statement

**Arturo Briseño-García:** Conceptualization, Methodology, Software, Writing – original draft.

## Declaration of Competing Interest

The authors declare that they have no known competing financial interests or personal relationships that could have appeared to influence the work reported in this paper.

## Data Availability

Board Interlocks, Network Characteristics and Corporate Social Responsibility data in the Mexican Stock Exchange (Original data) (Mendeley Data).

## Appendix Supplementary materials

Image, application 1
