# Supplementary material for: Board interlocks and Corporate Social Responsibility data in the Mexican Stock Exchange
Source: Data Brief. 2022 May 4;42:108231. doi: 10.1016/j.dib.2022.108231 (PMC9118658; doi:10.1016/j.dib.2022.108231)
Supplement: Supplementary file 1 [file mmc1.zip › Birds of a feather certify final.docx]

**Do birds of a feather certify together? The impact of board interlocks on CSR certification homophily**

**Abstract**

This study examines the homophily of Corporate Social Responsibility (CSR) practices among listed firms in the Bolsa Mexicana de Valores (Mexican Stock Exchange) (MSE). We draw upon concepts from institutional and social network theories to demonstrate how board interlocks among firms in the MSE can influence CSR practice adoption. Using a Mexican social and environmental certification, *Empresa Socialmente Responsable* (*ESR*), to represent the adoption of social and environmental practices and board interlocks among listed firms, we test hypotheses using multiple regression - quadratic assignment procedures (MR-QAP), which is relevant to the analysis of networks. Our main contribution is to show that mimetic, normative, and coercive institutional forces influence CSR adoption differently. Our results suggest that imitation *via* board interlocks is the most influential mechanism for CSR certification homophily among listed firms in Mexico. Normative pressures *via* industry affiliation are also influential, but only when combined with connections within the board interlock network. Our results provide insights into the wider diffusion of CSR practice.

**Keywords:**

Corporate Social Responsibility, Social Networks, Homophily, Institutional Theory

This research did not received any specific grant from funding agencies in the public, commercial, or not-for-profit sectors.

1. **Introduction**

Firms have increasingly adopted Corporate Social Responsibility (CSR) practices in light of continuous concerns from society regarding issues such as diversity, climate change, and poverty. The CSR literature explains the adoption of CSR practice by firms using both economic (Orlitzky, Schmidt, & Rynes, 2003; Jamali & Neville, 2011) and institutional perspectives (Matten & Moon, 2008). The economic reasons for CSR, also called “the business case,” have been tested with mixed results (e.g. McWilliams & Siegel, 2000; Zhao & Murrell, 2016). These studies have focused on finding a direct relationship between the firm’s engagement in social and environmental activities and its financial performance (Cavaco & Crifo, 2014; van Beurden & Gossling, 2008). Newer explanations for CSR adoption are based on institutional forces. This institutional perspective represents a sociological approach to CSR, which includes concepts such as imitation, norms and rules (DiMaggio & Powell, 1983).

While the economic reasons for CSR have been thoroughly tested with only mixed results (e.g. Barnett, 2007; McWilliams & Siegel, 2000; Zaho & Murrell, 2016), the institutional explanations for CSR have been more theoretical (e.g., Campbell, 2007) with less empirical research. More specifically, while practice adoption by the firm has been the focus of the CSR literature, to our knowledge, there are no attempts to understand CSR certification homophily, i.e. similarity between two firms with respect to a specific behavior – in this case – CSR certification. This similarity is important because CSR practices can be understood from a relational perspective where peer influence plays a greater role in practice adoption (Block, & Grund, 2014; Forman & van Zeebroeck, 2018; Lee & Qualls, 2010).

The relational aspect of CSR is especially important in Latin America, where awareness of the concept and adoption of practices can be influenced by interlocks. For example, Brazilian construction firm, Odebrecht appointed Sergio Foguel as a board member in charge of the compliance committee after serious allegations of corruption in the firm (Omics International, 2020). Foguel, with a long tradition as a board member in Obedrecht and other organizations (e.g. Heritage International Council and Dom Cabral Foundation), is actively involved in CSR activities on several boards. In Chile, Sudamericana de Valores, a logistic firm that uses containers for maritime transportation, appointed Christian Blomstrom as an independent board member in charge of the risk committee (“CMFChile”, 2020). Blomstrom is also an independent director in the Chilean retailer, La Polar, and is involved in corporate governance practices in both firms. These examples allow us to see the potential role of board interlocks in the adoption and diffusion of CSR practices.

Accordingly, the objective of this paper is to analyze relational influences in the homophilic adoption of CSR practices. In this study, we use a Mexican social and environmental certification, Empresa Socialmente Responsable (Socially Responsible Business) (*ESR*), which represents the presence of social and environmental practices in the adopters. Using board interlocks from listed firms in the Mexican Stock Exchange, we create a network of firms and test hypotheses using a Multiple Regression - Quadratic Assignment Procedure (MR-QAP).

The paper is structured as follows. First, we present relevant literature on CSR, institutional theory, and social networks. Second, we construct hypotheses around relational influences among listed firms and its impact on CSR adoption. Third, we describe the methodology used for the data collection and how this data was analyzed followed by an explanation of the results found in this study. Finally, we discuss the results and provide some concluding remarks.

1. **Literature Review**

A long standing, but still unresolved question in the CSR literature has been how a firm’s involvement in social and environmental activities affects its financial performance (Margolis and Walsh, 2003; van Beurden and Gössling, 2008). Less attention has been paid to the reasons why firms adopt CSR practices. Certainly, practice adoption may be due to the business case established by positive financial results, but adoption may also be due to non-financial reasons, which are postulated by institutional theory and network analysis.

**2.1 CSR and institutional theory**

The incorporation of institutional theory into the CSR literature represents an attempt by researchers to provide alternative explanations for practice adoption. In general, the institutional perspective concentrates on a social rather than a rational-economic logic based on a cost-benefit analysis for organizational action. Instead, the institutional literature holds that firms adopt practices based on taken-for-granted ideas, rather than for rational-economic reasons (DiMaggio & Powell, 1983).

Institutional theory is a relatively new theoretical framework for CSR with the potential to provide better explanations for CSR involvement (Brammer, Jackson, & Matten, 2012; Campbell, 2007; Hah & Freeman, 2013; Matten & Moon, 2008; Yang & Rivers, 2009). For example, Brammer, et al. (2008) state that institutional theory suits CSR because it helps to differentiate the paradox between voluntary engagement and the responsibilities imposed by society. Also, Matten & Moon (2008: 409) argue that institutional concepts serve well as a theoretical framework for CSR especially for what they call explicit CSR, which is defined as “corporate policies that assume and articulate responsibility for some societal interests.”

The main purpose behind the introduction of institutional theory into the CSR literature is to understand the role of the broader social and political context in the decision to engage in CSR practice, which is neglected in economic accounts. The key mechanism is isomorphism, which refers to the tendency of firms to adopt similar structures and practices. Isomorphism may be the outcome of mimetic, normative or coercive pressures. Mimetic isomorphism deals with the pressures firms experience to imitate the practices of other firms given uncertainty about the relationship between the means of CSR to achieve the ends of CSR (positive financial performance, political advantage, or even social well-being). In order to reduce uncertainty in their environment, firms will tend to adopt similar practices, thus resembling others. This modelling of practices, as DiMaggio and Powell (1983) call it, can happen through different channels, such as employee movement among firms, the use of consulting firms, participation in industry trade associations, or, in case of this paper, board interlocks.

In addition, the CSR literature has also examined mimetic isomorphism as a way to explain why firms adopt similar CSR practices. For example, Husted, Montiel, & Christmann (2016) analyze how MNC subsidiaries differ in adopting national or international social and environmental certifications. Focusing on MNCs in Mexico, they find that subsidiaries imitate nearby local firms, suggesting that international firms are also influenced by pressures for local legitimacy, creating local isomorphism.

Normative isomorphism refers to professionalization, which is “the collective struggle of members of an occupation to define the conditions and methods of their work” (DiMaggio & Powell, 1983, p. 150). Normative pressures emerge from two sources, one that relates to formal education and the other to professional associations. These factors create greater isomorphism of firms since employees receive similar training in universities and networks of professional associations help in the support and diffusion of similar practices.

Finally, coercive isomorphism “results from both formal and informal pressures exerted on organizations by organizations” (DiMaggio & Powell, 1983, p. 150). In the case of CSR practices, especially environmental practices, the regulations imposed by governments promote similar practices and structures among firms as they seek to comply with the law. In this study, we focus on mimetic, normative, and coercive isomorphism since it helps to explain the transmission of CSR practices among adopting firms.

**Social network theory and practice adoption**

Social network theory has gained momentum in organizational research, but less so in CSR. The limitations traditionally attributed to individual actors in a social environment and the increasing complexity of the organizational context has created the need for more complete answers instead of isolated explanations of the interactions in organizations (Borgatti & Foster, 2003).

Wellman (1983) defines social network analysis as the description of network patterns that limits social behavior and change. A network is defined as “a set of actors connected by a set of ties” (Borgatti & Foster, 2003 p.992). Each of these actors (nodes) can take the form of persons, teams or organizations, where the connections or ties among them are the central component for network theory. Ties, in comparison, consist of relationships of different types among actors ranging from friendships to shared drug use and from international trade to board interlocks.

To analyze the ties in a particular network, such as in the case of board interlocks, social network theory helps to explain how social interactions shape individual and organizational behavior (Wasserman & Faust, 2009). According to this literature, the ties among actors shape behavior and provide a richer understanding of the processes of social reality. As an emergent approach in social sciences, social network theory has evolved to become more robust in its methodology and more inclusive of other fields such as organizational studies, marketing science, and health studies (Rogers, 2003).

A particularly interesting characteristic of networks is homophily. This word comes from Greek roots that mean “together” (homo) and “friendship” or “love” (philos) and refers to the idea that people or groups with similar characteristics tend to interact more commonly. Hence the well-known aphorism, “Birds of a feather, flock together.” The concept has been extended to behavioral homophily, especially in the study of adolescent behavior (McPherson, Smith-Lovin, and Cook, 2001; Tsai, Valente, Miller, de la Haye, Pickering, and Cockburn, 2016). For example, Tsai, et al. (2016) discovered that ties among adolescents strongly influenced sun safety behavior, with closer relationships generating homophily in sun safety behavior. In this paper, we extend this literature to explain CSR certification homophily among firms based on board interlocks. By CSR certification homophily we mean the similarity of two firms in their adoption of a CSR certification.

The role of interlocks has been studied for many years as a particularly influential tie that connects firms (Mizruchi, 1996; Ornstein, 1984; Shropshire, 2010). An interlock is a director who participates in two or more corporate boards of directors and represents a potential conduit to transmit information from one firm to another (Davis & Greeve, 2007; Haunschild. 1993). A network of interlocks is the collection of dyadic relationships characterized by strong geographic dispersion in comparison to other types of networks (Ornstein, 1984). Here geographic dispersion means that the dyad can be formed by two people who could be residents of different cities, but see each other at board meetings at the same geographic location, i.e. the firm’s headquarters. This contrasts with other dyadic relationships with greater geographic proximity, such as classmates, friends, or employees.

The formation of board interlocks has been studied from different angles. For example, Mizruchi and Stearns (1988) present the traditional view that interlock creation is highly related to the firm’s capital dependence. This dependence is due to financial institutions that appoint representatives to the boards of firms with high long-term debt as a control mechanism.

The literature on interlocks is quite abundant and has evolved over the last decade, becoming much more specific, particularly, in understanding the processes that lead board members in different organizations to promote best practices (Mizruchi, 1996). However, research on interlocks has been highly concentrated in developed economies, perhaps because interlocks are more common in large public firms. An exception is the work of Rocha (2012), who analyzes board interlocks among public firms in Mexico.

Few studies in the CSR literature have focused on understanding the effect that network ties have on practice adoption (see, Davis & Greve, 1997; Galaskiewicz & Burt 1991; Shipilov, Greve, & Rowley, et al. 2010). However, the few studies that exist in the literature use traditional econometric methods to approach network data, reducing the explanatory power of relational data, which by their nature violate some critical assumptions of ordinary least squares, such as the independence of observations. For example, Ben Barka and Dandour (2015) study interlocks between groups using a network setting (interlocks) with traditional statistical analysis (ANOVA).

Clearly, the role of the social interactions is crucial in the adoption of practices across firms. Consequently, we take a social network perspective to approach CSR certification in Mexico.

1. **Hypotheses**

The main premise of this study is that social interactions among firms serve as conduits for CSR homophily. These conduits in the form of board interlocks, industry associations, or common business structures represent a way to test influence in a social system, particularly in the case of network ties (Mizruchi, 1996). As a result, this paper examines mimetic, normative, and coercive pressures using board interlocks, industry affiliation, and business group affiliation, respectively.

First, Haunschild & Beckman (1998) stress the importance of board interlocks as vehicles that facilitate communication and imitation, while Haunschild (1993) argues that interlocks are low-cost channels for the diffusion of information. The importance of board interlocks is centered not on their existence per se, but on the basic tie between two specific firms that the interlock represents (Mizruchi, 1996).

Actors in networks communicate information via direct contact (Galaskiewicz, & Burt, 1991). Direct contact is usually an effective way to transmit information since a face-to-face interaction is a strong mechanism for influence. For example, Simoni and Caiazza (2012) explain the formation of board interlocks for the type of information intended to be acquired by the firm. In their study, they found that firms establish certain ties with other firms, using interlocks, to obtain industry specific information. The authors suggest that this selective interlocking is used to gain access to knowledge that reinforces common industry practice, rather than new information. This is interesting in terms of the possible role that interlocks could play at later stages of diffusion where firms might use interlocks to maintain the status quo.

The direct contact mechanism is a cornerstone in network theory for organizational analysis since an organization’s decision making is not done in isolation, but embedded in a social system (Granovetter, 1985). The network ties, resulting from the interactions among organizations, help to communicate and diffuse ideas about CSR practice. As a result, a network approach can help trace the influence of board interlocks for dyadic practice adoption.

In this network context, contact with firms that have adopted certain practices creates the possibility of imitation under conditions of uncertainty (Attewell, 1992, Shipilov, et al. 2010). As the adoption spreads, the institutional pressure increases as a force that makes organizations comply with the *status quo* even though the practice often implies additional costs in its implementation (Meyer & Rowan, 1977). The mimetic mechanism explains how firms follow others by adopting similar practices based on direct interactions with others (DiMaggio & Powell, 1983). This is especially true when practices are uncertain in terms of either the benefits they produce or their implementation, a characteristic often found in CSR activities.

In terms of CSR practices, much of the discussion and the decision of how involved a business is in terms of social and environmental practices occurs at the top management level (Agle, Mitchell, & Sonnenfeld, 1999). This situation allows visibility of the practice for board members creating greater opportunities for exposure among those members that participate in two or more firms.

As a result, we expect that the network formed by board interlocks will help to communicate practices facilitating CSR certification homophily. As a result, we hypothesize:

*H1: The greater that the number of board interlocks shared by two firms, the greater the CSR certification homophily between them.*

Second, normative isomorphism occurs through a shared industry affiliation. Thus, industry similarity should also influence CSR certification homophily. The benchmarking that generally occurs within firms in the same industry creates opportunities for firms to become visible and similar in terms of managerial practices. Practices such as total quality management or *ISO 9000* can be found more frequently in one industry than others (Delmas & Montiel, 2008; Kennedy & Fiss 2009).

Firms in the same industry often participate in the same industry associations, which communicate similar practices among their members. Professional associations exert a form of normative isomorphism on their members (DiMaggio and Powell, 1983). This mechanism derives its major influence from professionalization, which is “the collective struggle of members of an occupation to define the conditions and methods of their work” (DiMaggio & Powell, 1983, p. 150). This definition of the conditions and methods of work emerges from two sources, one that relates to formal education and the other to professional associations. These factors create greater isomorphism of firms since employees receive similar training in universities and professional associations, which help support and diffuse similar practices (Galaskiewicz, 1985; Tolbert & Zucker, 1983).

As a result, a similar trait such as sharing the same industry will create greater communications channels and increase the likelihood that that those firms that have adopted the *ESR* certification will influence other firms in the same industry to adopt.

*H2: Firms of the same industry will be more likely to increase CSR certification homophily.*

Third, coercive pressure for practice adoption is often exerted through membership in the same business group. A business group is defined as those “individual firms that are associated by multiple links, potentially including cross ownership, close market ties, and social relations through which they coordinate to achieve mutual objectives” (Yiu, Lu, Bruton & Hoskisson, 2007 p.1551).

Business groups can mandate the adoption of CSR practices for all members of the same group (Cuervo-Cazurra, 2018). This pressure corresponds to coercive isomorphism as DiMaggio and Powell (1983) explain that firms become more similar because of the influence of authority. As a result, the inter-dependencies within business groups are an important factor to consider since they represent an important explanation for practice adoption (Guler, Guillén, & Macpherson 2002; Kostova & Roth, 2002).

Thus, it is expected that CSR certification homophily will be greater when the certifying firms belong to the same business group via coercion. As a result, we propose the following hypothesis:

*H3: Firms that are similar in terms of business group affiliation will be more likely to increase CSR certification homophily.*

As previously argued, the network ties, represented in this study with board interlocks, serve as vehicles for information sharing and uncertainty reduction (Srinivasan, Wuyts, & Mallapragada, 2018; Zona, Boyd, & Haynes, 2018). Furthermore, network ties can influence the adoption of practices among firms (Lamb & Roundy, 2016). Accordingly, since network ties are influential, regardless of the characteristics of peer firms, firms that share other common characteristics will likely exert greater influence on each other. For example, if a firm not only has interlocks with other firms, but also are in the same industry, the influence for CSR certification homophily will be greater. Hence, we hypothesize:

*H4: Firms of the same industry that also have board interlocks will increase CSR certification homophily.*

In the same way, membership in a business group can create greater influence for CSR certification homophily when firms not only belong to the same business group, but also are connected to other firms *via* board interlocks. The influence that the business group has over other members can create similarities among the members of the group (Morck & Yeung, 2003). For example, Khanna and Rivkin (2001) detect similarities in profitability among members of a business group, while Kim, Pae, and Yoo (2017) find that group-affiliated firms make more charitable contributions than non-affiliated groups. Thus, we hypothesize:

*H5: Firms that are part of a business group and have ties via board interlocks will increase CSR certification homophily.*

1. **Methodology**

Since the objective of this paper is to analyze the influence of network ties on CSR certification homophily, we collected information on CSR certification, the board of directors of firms listed in the Mexican Stock Exchange in 2011, and other control variables. We analyzed the data, using both methods that take into account the connected (non-independent) nature of the data as well as traditional methods.

**4.1 Dependent variable**

The *Empresa Socialmente Responsable* (*ESR*) certification is an initiative to recognize firms that engage in desirable social and environmental practices. We define certification as “the voluntary assessment and approval by an accredited party on an accredited standard” (Meuwissen et al., 2005:53). The *Centro Mexicano para la Filantropía* (*CEMEFI*) is the NGO responsible for the creation of the *ESR* certification in 2000. In order to get the certification, firms go through a process of self-evaluation in accordance with a set of pre-established guidelines. These guidelines comprise four areas, namely (1) quality of life, (2) business ethics, (3) relationships with the local community, and (4) environmental protection. After achieving a minimum level of social and environmental activity based on the self-evaluation and providing evidence for these activities, *CEMEFI* announces the newly certified firms and those that renew their previous certification. We use the 2012 *CEMEFI* list to find the firms that had been awarded the certification. As a result, by using adoption from 2012 and explanatory variables from 2011 we create a dynamic effect between dependent and independent variables. We do this following the suggestions of Cranmer, Leifeld, McClurg, & Rolfe (2017) for endogeneity, by manually incorporating dynamism in our model.

We use the *ESR* certification as a proxy for CSR practice adoption following the literature on the adoption of CSR certifications, which assumes that the certification reflects a wide range of individual practices and that on aggregate, it represents the firm’s involvement in CSR (Darnall & Sides, 2008; De Magistris et al.,2015; Husted et al., 2016). The CertificationESR_ij_ is our measure of CSR certification homophily and represents two listed firms of the network that have both adopted the *ESR* certification. If both firms *i* and *j* have adopted the certification, CertificationESR is scored “1”; if not, it is scored “0”.

**4.2 Independent variables**

***4.2.1 Board interlocks***

Firms listed in the Mexican Stock Exchange, following best practices in corporate governance, publish the names of their board members. To facilitate access to this information, the website of the Mexican Stock Exchange presents the annual reports, which contain the board members for all listed firms, including each position and type. In order to identify which person was a member of two or more boards, we created a list of the all the names in excel and then counted and marked each time a board member was on a different board.

Once collected, the initial 137 firms had a total of 2,171 directors registered. We then identified those board members who participated in more than one firm and filtered the names of directors alphabetically to eliminate repeated names. For each name, we indicate the different firms in which that person participates. The resulting matrix consists of 137 firms with 518 interlocks. Those interlocks were transformed into a *nxn* matrix with *X*_ij_ ties. Where n is the number of listed firms, i and j represent two listed firms of the network that have a tie based on a common board member. If *i* and *j* have a board interlock then *X* is “1”; if not, it is coded “0”.

***4.2.2 Industry***

The industry type was based on the classification of the stock exchange which divides firms into 10 types: (1) Energy, (2) Construction materials, (3) Industrial, (4) Service and non-basic goods, (5) Frequently consumed products, (6) Health, (7) Financial services, (8) Information Technologies, (9) Telecommunication services and (10) Public services. With this information, we created a *nxn* matrix with *X*_ij_ ties that represent industry similarity among the firms in the network, where *i* and *j* represent two firms of the network that have a tie based on operating in the same industry. So, if *i* and j belong to the same industry, then *X* is scored “1”; otherwise, it is scored “0”.

***4.2.3 Business groups***

For this study, business groups include firms that share cross-ownership, frequent economic transactions, and have strong relational links with other firms. Most of these firms are owned and managed by the same family with the exception of a few cases where the relationship is based on a subsidiary or other business unit. In the firms of this study, we identified 16 business groups. As a result, we created a *nxn* matrix with *X*_ij_ ties that indicate business group similarity, where *i* and *j* represent two firms of the network that belong to the same business group. In that case, *X* is scored with a “1”; otherwise, it is “0”.

**4.3 Control variables**

To control for firm characteristics, we use *Total Assets* to control for firm size, and *Return on Assets* *(ROA),* the ratio of profits divided by total firm assets to control for firm financial performance. This information was retrieved from Bloomberg. Using UCINET, we converted each firm observation into a *nxn* matrix with *X*_ij_ ties, where each tie represents size and performance similarity among the firms in the network. In this dyadic form, each variable expresses the financial similarity for that year. This is a continuous variable ranging from 0 to 1, where values near 1 represents greater similarity^[[1]](#footnote-1)^.

To control for network characteristics, we use Markov clustering and reach centrality. Markov clustering is a mathematical algorithm to detect groups of actors within a network structure (Stothert, Norberg, & Baugh, 2019). This network characteristic can influence practice adoption since it can detect sub-group influences within the network. Also, we control for reach centrality. Reach centrality is defined as the number of nodes that need to be crossed to get to another actor in the network (Radha, Kavikuil, & Keerthi, 2017). Reach is often described as walking a virtual path in the network resulting in a measure of distance. In this case, the longer the path, the smaller the centrality of a given actor in the network. Again, this network characteristic can influence practice adoption along with the actual network ties. Both metrics were calculated in UCINET and each firm’s metrics were converted into a *nxn* matrix with *X*_ij_ ties, where each tie represents the cluster and centrality similarity between each pair of firms in the network. In this dyadic form, each variable expresses the financial similarity for that year. This similarity is a continuous variable ranging from 0 to 1, where values near 1 represents greater similarity.

Finally, to control for CEO characteristics we use CEO duality and CEO power. The former occurs when the CEO is also the board chair. Controlling for CEO duality is important because the presence of the CEO on the board can serve to promote CSR activities (Jia, 2019). In fact, many social and environmental decisions are taken by the top management (Garriga & Melé, 2004; Waddock & Graves, 1997). The information about CEO duality was collected by analyzing public reports of each of the firms to detect those cases where the board chair also occupies the chief executive position. For listed Mexican firms, the same person occupies the role of CEO and board chair in around 40% of the firms. With this information, we created a *nxn* matrix with *X*_ij_ ties, where each tie represents CEO duality similarity between each pair of firms in the network. Where two firms of the network *i* and j both have a board chair who is also CEO, then *X* is scored with a “1”; otherwise, it is “0”.

CEO power in the firm was created using two individual variables, i.e., CEO duality and closeness centrality. The purpose is to create a variable at the firm level that represents the influence of the CEO in the network. First, as explained before, if the CEO is the chair of the board, there is a greater opportunity for that person to influence the adoption of practices; however, if the CEO is also from a central firm, that will increase the CEO’s power not only within the organization, but also within the network. As a result, we use closeness centrality to highlight CEO power in the network. We use Freeman’s closeness centrality, which represent the distance from a node to all remaining nodes in the network (Wasserman & Faust, 2009). With this information, we created a *nxn* matrix with *X*_ij_ ties, where each tie represents the similarity in closeness centrality between each pair of firms in the network. This matrix was then multiplied by the CEO duality matrix. The resulting *nxn* matrix indicates the centrality score of firms *i* and *j* only if the CEO is the board chair in both firms. All control variables are lagged one year to account for endogeneity in *ESR* adoption.

**4.4 Data analysis**

Board interlocks are common within business networks. However, despite the fact that board interlocks are used to create relational networks, the statistical methods frequently used to analyze them are based on assumptions of non-relational data (e.g., observations are assumed to be independent and identically distributed). Traditionally, management analyzes network ties using ordinary least squares (OLS), which loses explanatory power because it simplifies network ties into vectors containing only the number of interlocks (Briseño, Husted, & Rocha, 2019). Instead, this study uses MR-QAP as the regression approach to test hypotheses with network data. The econometric model in this paper is the following:

*CertificationESR*_ij_ *= β1 +β2 Interlocks*_ij_ *+ β3 Industry Sim*_ij_ *+ β4 Business Group Sim*_ij_ *+ β5 Interlocks x Industry_sim*_ij_ *+ β6 Interlocks x Business_Group_sim*_ij_ + α + *ε*

The purpose of social network analysis is to measure social interactions among actors that are related to and influenced by each other (Wasserman & Faust, 2009). As a result, econometric analysis for network relationships accounts for the dependence among observations, which is normally assumed to be absent for normal cross-sectional data with OLS. Clearly this is not the case with network data. The way out of the problem of non-independent observations is using the MR-QAP regression. The basic logic of MR-QAP is to provide a model with the observed data and then create permutations with the dependent variable to explain whether the observed model is significant. With 2000 permutations, MR-QAP creates a normal distribution for relational data.

Since MR-QAP requires a matrix to run the variables, we used the UCINET “attribute to matrix” command to convert the vector containing the information of the dependent and independent variables into matrices using the product option as the similarity metric. The product option generates an *nxn* matrix with ties *X*_ij_, where n is the number of listed firms in 2011, *X* is the tie or similarity and *i*, *j* are the two actors connected or not. In this model, we included a time lag to capture the effect of the interlocks in the adoption of the practice. As a result, the information for the dependent variable for CSR certification homophily corresponds to 2012.

1. **Results**

We statistically test the hypotheses in this this paper to determine the relational factors that increase CSR certification homophily. Table 1 presents the MR-QAP regressions with the CSR certification homophily as the dependent variable. Model 4 which includes all variables presents an R-square of 0.016 with the overall significance of 0.001. Low R-square values are generally associated with a low explanatory power of the statistical model. However, R-square values for MR-QAP regressions tend to be smaller and are not interpreted in the same manner as R-square in OLS regression (Gibbons, 2004; Krackhardt, 1988; Zagenczyk, Gibney, Few, & Purvis, 2013). The main reason for this difference resides in MR-QAP regression, which uses permutation-based hypothesis testing. Since permutations are created in order to randomly repeat combinations of rows and columns, MR-QAP cannot calculate statistical power or degrees of freedom (Ferrin, Dirks, & Shah, 2006). This means that correlations between two variables might be significant for one permutation, but not for other combinations of the same variables in subsequent permutations, hence the smaller valued of the R-square (Zagenczyk, et al. 2013).

Table 1. Regression results using OLS MR-QAP

| Dependent Variable = Similarity of ESR adoption (lagged 1 year) | | | | |  |  |  |  |  |  |  |  |  |
| --- | --- | --- | --- | --- | --- | --- | --- | --- | --- | --- | --- | --- | --- |
| Observations: 31152 |  |  |  |  |  |  |  |  |  |  |  |  |  |
| P-value Model1: 0.001 Rsq: 0.009 | Model 1 | | | Model 2 | | Model 3 | | Model 4 | | Model 5 | | Model 6 | |
| Intercept | 0.001 | *** |  | 0.001 | *** | 0.001 | *** | 0.001 | *** | 0.001 | *** | 0.001 | *** |
|  | (0.000) |  |  | (0.000) |  | (0.000) |  | (0.000) |  | (0.000) |  | (0.000) |  |
| Board Interlocks |  |  |  | 0.052 | *** | 0.051 | *** | 0.051 | *** | 0.024 |  | 0.048 | ** |
|  |  |  |  | (0.025) |  | (0.024) |  | (0.025) |  | (0.027) |  | (0.027) |  |
| Industry Similarity |  |  |  |  |  | 0.016 |  | 0.016 |  | 0.001 |  | -0.006 |  |
|  |  |  |  |  |  | (0.015) |  | (0.014) |  | (0.015) |  | (0.049) |  |
| Business Group Similarity |  |  |  |  |  |  |  | 0.008 |  | 0.009 |  | 0.004 |  |
|  |  |  |  |  |  |  |  | (0.041) |  | (0.041) |  | (0.041) |  |
| Board Interlocks x Industry Similarity |  |  |  |  |  |  |  |  |  | 0.064 | *** |  |  |
|  |  |  |  |  |  |  |  |  |  | (0.044) |  |  |  |
| Board Interlocks x Business Group Similarity |  |  |  |  |  |  |  |  |  |  |  | 0.009 |  |
|  |  |  |  |  |  |  |  |  |  |  |  | (0.047) |  |
| CEO-Power | 0.919 | *** |  | 0.897 | *** | 0.897 | *** | 0.896 | *** | 0.897 | *** | 0.898 | *** |
|  | (0.150) |  |  | (0.152) |  | (0.152) |  | (0.155) |  | (0.149) |  | (0.153) |  |
| CEO duality | -0.928 | *** |  | -0.906 | *** | -0.906 | *** | -0.904 | *** | -0.906 | *** | -0.907 | *** |
|  | (0.146) |  |  | (0.143) |  | (0.142) |  | (0.139) |  | (0.142) |  | (0.146) |  |
| Reach centrality | 0.047 | * |  | 0.044 | * | 0.042 |  | 0.041 | * | 0.041 |  | 0.044 | * |
|  | (0.028) |  |  | (0.029) |  | (0.029) |  | (0.029) |  | (0.029) |  | (0.028) |  |
| Clusters | 0.046 | ** |  | 0.037 | * | 0.037 | * | 0.036 | * | 0.035 | * | 0.036 | * |
|  | (0.034) |  |  | (0.034) |  | (0.034) |  | (0.033) |  | (0.033) |  | (0.033) |  |
| ROA | 0.045 |  |  | 0.045 |  | 0.045 |  | 0.045 |  | 0.047 |  | 0.045 |  |
|  | (0.024) |  |  | (0.024) |  | (0.024) |  | (0.024) |  | (0.024) |  | (0.024) |  |
| Total Assets | -0.049 | * |  | -0.048 | * | -0.049 | * | -0.049 | ** | -0.049 | ** | -0.049 | * |
|  | (0.032) |  |  | (0.030) |  | (0.031) |  | (0.031) |  | (0.030) |  | (0.031) |  |
| ***p<0.01, **p<0.05, *p<0.1 |  |  |  |  |  |  |  |  |  |  |  |  |  |

As presented in models 2 to 6, H1 is positive and significant. Model 2 presents a coefficient of β2 = 0.052 (p<0.01), meaning that having a tie via board interlocks significantly influences CSR certification homophily. These results show that connections with other firms matter for CSR certification homophily, suggesting that the institutional mechanism involved in direct contact might be mimetic isomorphism.

As presented also in Table 1, H2 is not significant. Belonging to the same industry does not influence CSR certification homophily. In this case, the normative mechanism does not increase CSR certification homophily among its members. A possible explanation of this result is the fact that the sample in this study is limited to those firms that are listed in the Mexican Stock Exchange. As a result, each firm’s industry network extends beyond the sample, leaving out other firms in the same industry. This situation may cause the lack of explanatory power in this variable. Also, the characteristic of the practice can be a factor in terms of industry. Since *ESR* is a general certification, not specific to some industries such as the case of Forest Stewardship Council (FSC) certification for sustainably forested lumber, the industry might not be a relevant factor influencing practice adoption.

Hypothesis 3, which tests the influence of Business Groups in CSR certification homophily, is also not significant. Firms that belong to the same business group do not increase CSR certification homophily. Again, the fact that listed firms might represent only a proportion of the members of the business group could explain the apparent lack of a relationship between CSR certification homophily and belonging to the same business group. Other explanations might be related to the very complex relationships around business groups in Mexico. For example, the market pressures and family ties that exist in many business groups can also be predictors of coercive pressures for similarity of *ESR* adoption.

In order to test the extent of the influence of network ties (board interlocks) and under what conditions the industry and the business group can be influential, we test the interaction among these variables. Model 5 presents hypothesis 4, which includes the interaction between board interlocks and industry. The coefficient of β5 = 0.064 (p<0.01), means that ties *via* board interlocks with other firms from the same industry increases CSR certification homophily. This result suggests that industry similarity alone is not enough to explain CSR certification homophily, but the combination of firms in the same industry with a board interlock does increase the likelihood of CSR certification.

Also, we test hypothesis 5 in model 6. The coefficient of β6 = 0.009 (p>0.1), means that ties *via* board interlocks with other firms from the same business group does not influence CSR certification homophily. As a result, the coercive mechanism within the business group does not change even when direct contact exists between two firms.

**5.1 Instrumental variable regression**

We used an instrumental variable strategy recommended for detecting and correcting potential endogeneity (Wooldridge. 2016). Since the instrumental variable approach cannot be applied with MQAP, we follow Chellapa and Saraf (2010) and Maroofi (2012) to employ an OLS regression to test for endogeneity. In our model, potential endogeneity may arise from the possibility that CSR could be part of the criteria used for board interlock formation (Breneke and Rank, 2017). Given that we focus on the first independent variable as the endogenous variable, our goal is to find an instrumental variable, i.e., a variable Z that is correlated with the assumed endogenous variable, in this case board interlocks, but uncorrelated with the disturbances of the error term (Greene, 2012). Accordingly, we used the clusters variable as an instrument for board interlocks. This variable represents the focal firm’s position in the network, calculated using a Markov algorithm to detect groups of actors within a network structure (Stothert, Norberg, & Baugh, 2019). A firm’s position in the network should not be related to the endogenous problem from board selection and CSR adoption, while remaining a proxy for network influence over CSR adoption. Since the Wu-Hausman F test for endogeneity is not significant (p>0.1), clusters can be used as an instrument for our model.

After determining that Clusters is a valid instrument for the model, we use this variable in an OLS regression with CSR adoption as the dependent variable. The clusters variable is significant at p<.000, confirming the first hypothesis. As a result, the use of board interlocks appears to be valid in our model.

**Discussion and Conclusion**

This paper presents relational factors that influence CSR practices. Using *ESR*, a social and environmental certification in Mexico, this study shows that board interlocks and membership in the same business group influences CSR certification homophily. Our results help to explain how firms can influence peer firms to adopt CSR practices, i.e., increasing firm's connections can create greater communication channels for firms in terms of social and environmental practices.

Our results also show that the major factor influencing CSR certification homophily among listed firms is direct contact via board interlocks. The board members that link two or more firms influence CSR certification homophily. The more direct contact (i.e. board interlocks) with other firms creates more opportunities to imitate those firms that have adopted the *ESR* certification. Due to the increasing demands from society for social and environmental activities, firms look for benchmarks among peer firms to diminish the uncertainty involved in adopting practices that are, to some extent, unknown to them. Since CSR has multiple definitions and includes a wide spectrum of activities, firms in MSE may use peer benchmarks for the adoption of the *ESR* certification.

The influence of industry and business groups in our study does not show an effect on CSR practice adoption. The literature shows clear evidence that belonging to an industry supports the adoption of practices (Cheng, 2010; Gondo & Amis, 2013; O´Connor, Parcha & Tulibaski, 2017). However, in our study, industry similarity does not have explanatory power since CSR practice adoption is not uniquely related to industry characteristics. For example, O'Connor et al. (2017) show that groups of companies in the same industry have different characteristics, so that CSR adoption is due to other factors such as the relationship of stakeholders (companies), the level of existing information within and across industries (Mukherjee & Muga, 2010), or the requirements to adopt a practice (Rodríguez, Cuadrado, Martínez & García, 2017).

As a result, belonging to the same industry does not guarantee that firms adopt the same practice in the network of listed firms in this study. This result implies that, for Mexican firms, the socialization of practices is stronger via direct contact than through industry membership as institutional theory suggests. This result could be due to the non-compulsory nature of the *ESR* certification. Many industries have compulsory certifications that are promoted among their members (Abrahamson, & Fairchild, 1999; King, Lenox, & Terlaak, 2005). Since *ESR* certification, similar to *ISO 14000*, is not compulsory, the industry normative mechanism does not provide explanatory power.

In the case of business groups, our results show that business group similarity does not influence *ESR* certification adoption. As in the industry case, the influence from business association in the network does not influence adoption as the literature suggests. A possible explanation of our results is the involvement of business groups in an emerging economy such as Mexico. Cuervo-Cazurra (2018) suggests that business group involvement in CSR investments can change depending on the level of economic development in each country. He notes that business groups in emerging markets, compared to other developing economies, invest less in CSR since governments are more involved in or capable of attending to social needs, making the adoption of CSR certification less attractive (Cuervo-Cazurra, 2018).

In addition, we test the interaction of board interlocks in the specific cases of both industry and business groups. We find positive results for CSR certification homophily and industry similarity, suggesting that it is not the similarity in the industry alone, but the connections between firms that increase CSR certification homophily. This result suggests that CSR practices can be increased when firms are in the same industry and share board members. As a result, we contribute to the CSR literature by testing institutional factors influencing CSR practice adoption that can be studied and go beyond the classical reasons related to firm performance.

The fact that network ties matter is also a relevant contribution in this study. Specifically, we contribute to the management literature by suggesting that not all social contexts in which a firm is immersed increase practice adoption. In this study, the normative and coercive mechanisms do not influence the adoption of the *ESR* certification. However, the normative mechanism via membership in the same industry does have an influence when firms also share a board interlock. We also contribute to the literature by using social network data and models such as MR-QAP, rarely seen in the management literature. By using social network analysis, researchers can increase the study of organizations considering the relational and institutional aspects in which organizations are immersed.

The Mexican context used in this study is interesting for policy making in terms of practice adoption, especially around social and environmental issues. In particular, the highly relational characteristics that are presented in developing countries, such as the appointment of shared board members among listed firms, can be important to promote CSR practices and target firms to increase CSR adoption and help in solving some of the social and environmental problems present in these regions. Also, understanding the factors affecting the firm’s involvement in CSR certification is important to improve awareness of the CSR concept and help to standardize practices across firms. Firms without the certification could be involved in CSR practices, but not benefit from the knowledge or experience that comes from a third-party evaluation and continuous improvement of CSR practices.

Some limitations exist in the elaboration of this paper with respect to the time and place of the study as well as the direction of ties. First, the data is taken for a single year which does not reflect changes or relationships over time. Further research should consider the changes in tie formation longitudinally. Even though the dependent variable is lagged one year, the current study takes a cross-sectional approach. By designing a longitudinal study, researchers can create better explanations for CSR certification homophily and the adoption and diffusion of CSR practices.

In addition, the study is limited to the Mexican context. However, as the cases of Obedrecht in Brazil and Sudamericana de Valores in Chile indicate, the potential influence of board interlocks on CSR adoption exists in other Latin American countries. Hence it would be important to extend this study to other countries in order to understand the potential and limits of board interlocks as a vehicle for spreading socially responsible practices in other contexts.

Finally, in terms of tie direction, this paper uses undirected networks, which implies a symmetric relationship between any two pair of firms. Although literature using undirected networks at the firm level is common (Borgatti, et al. 2011), we suggest that future research should attempt to provide additional information on the directionality of relationships for CSR adoption. Finally, further research should analyze different networks that consider firms other than publicly traded ones.

Our results help to explain how board interlocks created by firms can influence peers to adopt homophilic CSR practice. The highly relational characteristics that are presented in developing countries can be important to promote CSR practices strategically and effectively target firms to increase CSR adoption.

1. **References**

Abrahamson, E., & Fairchild, G. (1999). Management fashion: Lifecycles, triggers, and collective learning processes. *Administrative science quarterly*, 44(4), 708-740. https://doi.org/10.2307/2667053

Agle, B. R., Mitchell, R. K., & Sonnenfeld, J. A. (1999). Who matters to CEOs? An investigation of stakeholder attributes and salience, corpate performance, and Ceo values.*Academy of Management Journal*, 42(5), 507-525. https://doi.org/10.5465/256973

Attewell, P. (1992). Technology diffusion and organizational learning: The case of business computing.*Organization Science*, 3(1), 1-19. https://doi.org/10.1287/orsc.3.1.1

Barnett, M. L. (2007). Stakeholder influence capacity and the variability of financial returns to corporate social responsibility.*Academy of Management Review*, 32(3), 794-816. https://doi.org/10.2307/20159336

Ben Barka, H. & Dardour, A. (2015). Investigating the relationship between director’s profile, board interlocks and corporate social responsibility. *Management Decision,* 53(3), 553-570. https://doi.org/10.1108/MD-12-2013-0655

Biography Global Scientist. Business and Management Experts, (2020, October 3). Retrieved from: https://biography.omicsonline.org/brazil/odebrecht/sergio-foguel-1266296.

Block, P., & Grund, T. (2014). Multidimensional homophily in friendship networks. *Network Science*, 2(2),189-212. https://doi.org/10.1017/nws.2014.17

Borgatti, S. P., Mehra, A., Brass, D. J., & Labianca, G. (2009). Network analysis in the social sciences. *Science,* 323(5916), 892-895. https://doi.org/10.1126/science.1165821

Borgatti, S.P., & Foster, P.C. (2003). The network paradigm in organizational research: A review and typology. *Journal of Management*, 29 (6). 991–1013. https://doi.org/10.1016/S0149-2063(03)00087-4.

Brammer, S., Jackson, G., & Matten, D. (2012). Corporate social responsibility and institutional theory: New perspectives on private governance.*Socio-economic Review*, 10(1), 3-28. https://doi.org/10.1093/ser/mwr030

Brennecke, J., & Rank, O. N. (2017). Tie heterogeneity in networks of interlocking directorates: a cost–benefit approach to firms’ tie choice. *Business Research*, 10(1), 97-122. https://doi.org/10.1007/s40685-016-0042-7

Briseño, A., Husted, B. W., & Rocha, J. M. (2019). Methodological problems on the diffusion of managerial practices. *Contaduría y Administración*, *64*(1), 1-15. E-Publishing Inc.
Reference to a website: https://ideas.repec.org/a/nax/conyad/v64y2019i1p11-12.html

Campbell, J. L. (2007). Why would corporations behave in socially responsible ways? An institutional theory of corporate social responsibility.*Academy of Management Review,*  32 (3), 946-967. https://doi.org/10.2307/20159343

Cavaco, S., & Crifo, P. (2014). CSR and financial performance: Complementarity between environmental, social and business behaviours.*Applied Economics*, 46(27), 3323-3338. https://doi.org/10.1080/00036846.2014.927572

Chellappa, R. K., & Saraf, N. (2010). Alliances, rivalry, and firm performance in enterprise systems software markets: a social network approach. *Information Systems Research*, *21*(4), 849-871. https://doi.org/10.1287/isre.1090.0278

Cheng, H. L. (2010). Seeking knowledge or gaining legitimacy? Role of social networks on new practice adoption by OEM suppliers. *Journal of Business Research*, 63(8), 824-831. https://doi.org/10.1016/j.jbusres.2009.09.023

CMFChile, (2020, Octubre, 3). Comisión para el Mercado Financiero. Retrieved from: http://www.cmfchile.cl/institucional/mercados/entidad.php?mercado=V&rut=96874030&grupo=0&tipoentidad=RVEMI&row=&vig=VI&control=svs&pestania=49.

Cranmer, S. J., Leifeld, P., McClurg, S. D., & Rolfe, M. (2017). Navigating the range of statistical tools for inferential network analysis. *American Journal of Political Science,* 61(1), 237-251. https://doi.org/10.1111/ajps.12263

Cuervo-Cazurra, A. (2018). The evolution of business groups’ corporate social responsibility. *Journal of Business Ethics*, 153(4), 997-1016. https://doi.org/10.1007/s10551-018-3912-4

Darnall, N., & Sides, S. (2008). Assessing the performance of voluntary environmental programs: does certification matter?. *Policy Studies Journal*, 36(1), 95-117. https://doi.org/10.1111/j.1541-0072.2007.00255.x

Davis, G. and Greve, H.R. (1997). Corporate elite networks and governance changes in the 80’s. *American Journal of Sociology*, 103 (1), 1-37. https://doi.org/10.1086/231170

De Magistris, T., Del Giudice, T., & Verneau, F. (2015). The effect of information on willingness to pay for canned tuna fish with different corporate social responsibility (CSR) certification: a pilot study.*Journal of Consumer Affairs*, 49 (2), 457-471. https://doi.org/10.1111/joca.12046

Delmas, M., & Montiel, I. (2008). The diffusion of voluntary international management standards: Responsible Care, ISO 9000, and ISO 14001 in the chemical industry.*Policy Studies Journal,* 36 (1), 65-93. https://doi.org/10.1111/j.1541-0072.2007.00254.x

DiMaggio, P. J., & Powell, W. W. (1983). The iron cage revisited: Institutional isomorphism and collective rationality in organizational fields. *American Sociological Review,* 147-160. https://doi.org/10.2307/2095101

Ferrin, D. L., Dirks, K. T., & Shah, P. P. (2006). Direct and indirect effects of third-party relationships on interpersonal trust.*Journal of Applied Psychology,* 91 (4), 870. https://doi.org/10.1037/0021-9010.91.4.870

Forman, C., & van Zeebroeck, N. (2018). Digital technology adoption and knowledge flows within firms: Can the Internet overcome geographic and technological distance?*Research Policy*, 47 (4). https://doi.org/10.1016/j.respol.2018.10.021

Galaskiewicz, J. (1985). Interorganizational relations.*Annual Review of Sociology*, 11(1), 281-304. https://doi.org/10.1146/annurev.so.11.080185.001433

Galaskiewicz, J., & Burt, R. S. (1991). Interorganization contagion in corporate philanthropy.*Administrative Science Quarterly*, 36 (1), 88-105. https://doi.org/10.2307/2393431

Garriga, E., & Melé, D. (2004). Corporate social responsibility theories: Mapping the territory*. Journal of Business Ethics,* 53 (2), 51-71. https://doi.org/10.1023/B:BUSI.0000039399.90587.34

Gibbons, D. E. (2004). Friendship and advice networks in the context of changing professional values.*Administrative Science Quarterly*, 49 (2), 238-262. https://doi.org/10.2307/4131473

Gondo, M. B., & Amis, J. M. (2013). Variations in practice adoption: The roles of conscious reflection and discourse. *Academy of Management Review*, 38 (2), 229-247. https://doi.org/10.5465/amr.2010.0312

Granovetter, M. (1985). Economic action and social structure: The problem of embeddedness. *The American Journal of Sociology*, 91 (3), 481-510. https://doi.org/10.2307/2780199.

Greene, W. H. (2012). *Econometric analysis*. Pearson Education Upper Saddle River USA.

Guler, I., Guillén, M. F., & Macpherson, J. M. (2002). Global competition, institutions, and the diffusion of organizational practices: The international spread of ISO 9000 quality certificates.*Administrative Science Quarterly,* 47 (2), 207-232. https://doi.org/10.2307/3094804

Haunschild, P. R. (1993), Interorganizational imitation: The impact of interlocks on orporate acquisition activity. *Administrative Science Quarterly*, 38 (4), 564. https://doi.org/10.2307/2393337

Haunschild, P. R., & Beckman, C. M. (1998). When do interlocks matter?: Alternate sources of information and interlock influence.*Administrative Science Quarterly, 43 (4),* 815-844. https://doi.org/10.2307/2393617

Hofstede, G. (1984). The cultural relativity of the quality of life concept.*Academy of Management review*, 9 (3), 389-398. https://doi.org/10.2307/258280

Husted, B. W., Montiel, I., & Christmann, P. (2016). Effects of local legitimacy on certification decisions to global and national CSR standards by multinational subsidiaries and domestic firms.*Journal of International Business Studies*, 47(3), 382-397. https://doi.org/10.1057/jibs.2016.3

Jamali, D., & Neville, B. (2011). Convergence versus divergence of CSR in developing countries: An embedded multi-layered institutional lens.*Journal of Business Ethics,* 102 (4), 599-621. https://doi.org/10.1007/s10551-011-0830-0

Jia, X. (2020). Corporate social responsibility activities and firm performance: The moderating role of strategic emphasis and industry competition. *Corporate Social Responsibility and Environmental Management*, *27* (1), 65-73. https://doi.org/10.1002/csr.1774

Kennedy, M. T., & Fiss, P. C. (2009). Institutionalization, framing, and diffusion: The logic of TQM adoption and implementation decisions among US hospitals.*Academy of Management Journal,* 52 (5), 897-918. https://doi.org/10.5465/amj.2009.44633062

Khanna, T., & Rivkin, J. W. (2001). Estimating the performance effects of business groups in emerging markets.*Strategic Management Journal*, 22(1), 45-74. https://doi.org/10.1002/1097-0266(200101)22:1%3C45::AID-SMJ147%3E3.0.CO;2-F

Kim, B., Pae, J., & Yoo, C. Y. (2019). Business groups and tunneling: Evidence from corporate charitable contributions by Korean companies.*Journal of Business Ethics*, 154 (3), 643-666. https://doi.org/10.1007/s10551-016-3415-0

King, A. A., Lenox, M. J., & Terlaak, A. (2005). The strategic use of decentralized institutions: Exploring certification with the ISO 14001 management standard. *Academy of management journal*, 48 (6), 1091-1106. https://doi.org/10.5465/amj.2005.19573111

Kostova, T., & Roth, K. (2002). Adoption of an organizational practice by subsidiaries of multinational corporations: Institutional and relational effects. *Academy of Management Journal,* 45 (1), 215-233. https://doi.org/10.2307/3069293

Lamb, N. H., & Roundy, P. (2016). The “ties that bind” board interlocks research: a systematic review.*Management Research Review*, 39 (11), 1516-1542. https://doi.org/10.1108/MRR-02-2015-0027

Lee, J., & Qualls, W. J. (2010). A dynamic process of buyer-seller technology adoption.*Journal of Business & Industrial Marketing*, 25 (3), 220-228. https://doi.org/10.1108/08858621011027812

Maroofi, F. (2012). Network structure and network effects and consumer interaction in mobile telecommunications among students. *African Journal of Marketing Management*, 4 (2), 55-64. https://doi.org/10.5897/AJMM11.083

Matten, D., & Moon, J. (2008). “Implicit” and “explicit” CSR: A conceptual framework for a comparative understanding of corporate social responsibility.*Academy of Management Review,* 33 (2), 404-424. https://doi.org/10.2307/20159405

McPherson M, Smith‐Lovin L, & Cook JM. (2001). Birds of a Feather: Homophily in Social Networks. *Annual Review of Sociology*. 27 (1), 415‐444. https://doi.org/10.1146/annurev.soc.27.1.415

McWilliams, A., & Siegel, D. (2000). Corporate social responsibility and financial performance: correlation or misspecification?.*Strategic Management Journal*, 21 (5), 603-609. https://doi.org/10.1002/(SICI)1097-0266(200005)21:5%3C603::AID-SMJ101%3E3.0.CO;2-3

Meyer, J. W., & Rowan, B. (1977). Institutionalized organizations: Formal structure as myth and ceremony.*American Journal of Sociology*, 83 (2), 340-363. https://doi.org/10.1086/226550

Meuwissen, M. P., Velthuis, A. G., Hogeveen, H., & Huirne, R. B. (2003). Technical and economic considerations about traceability and certification in livestock production chains. *New approaches to food safety economics*, Wageningen, 41-54.

Mizruchi, M. S. (1996). What do interlocks do? An analysis, critique, and assessment of research on interlocking directories. *Annual Review of Sociology*, 22 (1), 271-293. http://dx.doi.org/10.1146/annurev.soc.22.1.271

Mizruchi, M. S., & Stearns, L. (1988), A longitudinal study of the formation of interlocking directorates. *Administrative Science Quarterly*, 33 (2), 194-221. http://dx.doi.org/10.2307/2393055.

Morck, R., & Yeung, B. (2003). Agency problems in large family business groups.*Entrepreneurship Theory and Practice*, 27 (4), 367-382. https://doi.org/10.1111/1540-8520.t01-1-00015

Mukherjee, A., & Muga, H. (2010). An integrative framework for studying sustainable practices and its adoption in the AEC industry: A case study. *Journal of Engineering and Technology Management*, 27 (3-4), 197-214. http://doi.org/10.1016/j.jengtecman.2010.06.006

O’Connor, A., Parcha, J. M., & Tulibaski, K. L. (2017). The institutionalization of corporate social responsibility communication: An intra-industry comparison of MNCs’ and SMEs’ CSR reports. *Management Communication Quarterly*, 31 (4), 503-532. https://doi.org/10.1177/0893318917704512

Orlitzky, M., Schmidt, F. L., & Rynes, S. L. (2003). Corporate social and financial performance: A meta-analysis.*Organization Studies*, 24 (3), 403-441. https://doi.org/10.1177/0170840603024003910

Ornstein, M. (1984), Interlocking Directorates in Canada: Intercorporate or Class Alliance?. *Administrative Science Quarterly,* 29 (2), 210-231. https://doi.org/10.2307/2393174

Radha, D., Kavikuil, K., & Keerthi, R. (2017). Centrality Measures to Analyze Transport Network for Congestion Free Shipment. *In 2017 2nd International Conference on Computational Systems and Information Technology for Sustainable Solution (CSITSS)*,1-5. https://doi.org/10.1109/CSITSS.2017.8447765

Rocha, J. M. (2012), Business groups as hierarchical clique structures: A conceptual and methodological discussion as it applies to the Mexican experience. *British Journal of Management,* 23 (3), 291-306. https://doi.org/10.1111/j.1467-8551.2011.00740.x

Rodríguez‐Ariza, L., Cuadrado‐Ballesteros, B., Martínez‐Ferrero, J., & García‐Sánchez, I. M. (2017). The role of female directors in promoting CSR practices: An international comparison between family and non‐family businesses. *Business Ethics: A European Review,* 26 (2), 162-174. https://doi.org/10.1111/beer.12140

Rogers, E. M. (2003). *Diffusion of innovations*. Free Press, N.Y.

Shipilov, A. V., Greve, H. R., & Rowley, T. J. (2010). When do interlocks matter? Institutional logics and the diffusion of multiple corporate governance practices.*Academy of Management Journal*, 53 (4), 846-864. https://doi.org/10.5465/amj.2010.52814614

Shropshire, C. (2010), The role of the interlocking director and board receptivity in the diffusion of practices. *Academy of Management Review*, 35 (2), 246-264. https://doi.org/10.5465/AMR.2010.48463333.

Simoni, M. M., & Caiazza, R. R. (2012). How does learning intent affect interlocking directorates dynamic? *Learning Organization*, 19 (5), 388-399. https://doi.org/10.1108/09696471211239695.

Srinivasan, R., Wuyts, S., & Mallapragada, G. (2018). Corporate board interlocks and new product introductions.*Journal of Marketing,* 82 (1), 132-148. https://doi.org/10.1509/jm.16.0120

Stothert, L., Norberg, P., & Baugh, C. M. (2019). A new approach to finding galaxy groups using Markov Clustering. *Monthly Notices of the Royal Astronomical Society: Letters,* 485 (1), 126-130. https://doi.org/10.1093/mnrasl/slz045

Tsai, J., Valente, T. W., Miller, K. A., De La Haye, K., Pickering, T. A., & Cockburn, M. G. (2016). Friendship networks and sun safety behavior among children.*Network Science,*4 (3), 314-335. https://doi.org/10.1017/nws.2016.6

Van Beurden, P., & Gössling, T. (2008). The worth of values–a literature review on the relation between corporate social and financial performance. *Journal of Business Ethics,* 82 (2), 407-424. https://doi.org/10.1007/s10551-008-9894-x

Waddock, S. A., & Graves, S. B. (1997). The corporate social performance-financial performance link.*Strategic Management Journal, 18* (4), 303-319. https://doi.org/10.1002/(SICI)1097-0266(199704)18:4%3C303::AID-SMJ869%3E3.0.CO;2-G

Wasserman, S., & Faust, K. (2009). *Social Network Analysis: Methods and Applications.* Cambridge: Cambridge University Press.

Wellman, B. (1983). Network analysis: Some basic principles. *Sociological Theory*, 1 (1), 155-200. https://doi.org/10.2307/202050

Wooldridge, J. M. (2016). *Introductory econometrics: A modern approach*. Nelson Education.

Yiu, D. W., Lu, Y., Bruton, G. D., & Hoskisson, R. E. (2007). Business groups: An integrated model to focus future research.*Journal of Management Studies*, 44 (8), 1551-1579. https://doi.org/10.1111/j.1467-6486.2007.00735.x

Zagenczyk, T. J., Gibney, R., Few, W. T., & Purvis, R. L. (2013). The ties that influence: A social network analysis of prototypical employees' effects on job attitudes among coworkers.*Journal of Management Policy and Practice*, 14 (4), 26-42. https://doi.org/10.1111/j.1467-6486.2007.00735.x

Zhao, X., & Murrell, A. J. (2016). Revisiting the corporate social performance‐financial performance link: A replication of W addock and G raves.*Strategic Management Journal,* 37 (11), 2378-2388. https://doi.org/10.1111/j.1467-6486.2007.00735.x

1. For our model, we need to transform a financial metric such as Total Assets and ROA from a node value to a dyadic value (a metric that represent similarity between two firms). As a result, we transformed the economic value of total assets and the ratio representing the economic value of profits divided by the economic value of total assets into a dyadyc metric to form a similarity matrix. We use UCINET’s attribute-to-matrix command using the identity coefficient option. The resulting matrix represents a coefficient ranging from 0 to 1 explaining how similar two firms are in terms two nodal characteristics, i.e. Total Assets and ROA. An identity coefficient near one represents greater similarity, while an identity coefficient near zero represents lower similarity between two firms. [↑](#footnote-ref-1)
