# Supplementary material for: Board interlocks and Corporate Social Responsibility data in the Mexican Stock Exchange
Source: Data Brief. 2022 May 4;42:108231. doi: 10.1016/j.dib.2022.108231 (PMC9118658; doi:10.1016/j.dib.2022.108231)
Supplement: Supplementary file 1 [file mmc1.zip › Title page.docx]

**Do birds of a feather certify together? The impact of board interlocks on CSR certification homophily**

Arturo Briseño

Universidad Autónoma de Tamaulipas

Facultad de Comercio y Administración Victoria

Matamoros S/N Zona Centro, Cd. Victoria, Tamaulipas, México

Ph. +52-834-145-1303

abriseno@docentes.uat.edu.mx

Bryan William Husted

EGADE Business School

Tecnológico de Monterrey

Av. Eugenio Garza Lagüera and Rufino Tamayo s/n

Valle Oriente, Garza García, N.L., 66269 México

Ph. +52-81-8625-6150

bhusted@tec.mx

Eduardo Arango-Herera

*Corresponding author*

Universidad Autónoma de Tamaulipas

Facultad de Comercio y Administración Victoria

Matamoros S/N Zona Centro, Cd. Victoria, Tamaulipas, México

Ph. +52-834-145-1303

earango@docentes.uat.edu.mx
